# Cognitive and neural consequences of memory suppression in major depressive disorder

**DOI:** 10.3758/s13415-016-0464-x

**Published:** 2016-09-20

**Authors:** Matthew D. Sacchet, Benjamin J. Levy, J. Paul Hamilton, Arkadiy Maksimovskiy, Paula T. Hertel, Jutta Joormann, Michael C. Anderson, Anthony D. Wagner, Ian H. Gotlib

**Affiliations:** 1 Neurosciences Program, Stanford University, Stanford, CA USA; 2 Department of Psychology, Stanford University, Jordan Hall, Building 01-420, 450 Serra Mall, Stanford, CA 94305 USA; 3 Translational Research Center for TBI and Stress-Related Disorders (TRACTS), VA Boston Healthcare System, Boston, MA USA; 4 Department of Psychology, University of San Francisco, San Francisco, CA USA; 5 Center for Social and Affective Neuroscience, Department of Clinical and Experimental Medicine, Linköping University, Linköping, Sweden; 6 Department of Behavioral Neuroscience, Boston University School of Medicine, Boston, MA USA; 7 Department of Psychology, Trinity University, San Antonio, TX USA; 8 Department of Psychology, Yale University, New Haven, CT USA; 9 MRC Cognition and Brain Sciences Unit, University of Cambridge, Cambridge, UK

**Keywords:** Major depressive disorder (MDD), Functional magnetic resonance imaging (fMRI), Memory suppression, Think/No-Think (TNT), Cognitive neuroscience, Negative valence

## Abstract

Negative biases in cognition have been documented consistently in major depressive disorder (MDD), including difficulties in the ability to control the processing of negative material. Although negative information-processing biases have been studied using both behavioral and neuroimaging paradigms, relatively little research has been conducted examining the difficulties of depressed persons with inhibiting the retrieval of negative information from long-term memory. In this study, we used the think/no-think paradigm and functional magnetic resonance imaging to assess the cognitive and neural consequences of memory suppression in individuals diagnosed with depression and in healthy controls. The participants showed typical behavioral forgetting effects, but contrary to our hypotheses, there were no differences between the depressed and nondepressed participants or between neutral and negative memories. Relative to controls, depressed individuals exhibited greater activity in right middle frontal gyrus during memory suppression, regardless of the valence of the suppressed stimuli, and differential activity in the amygdala and hippocampus during memory suppression involving negatively valenced stimuli. These findings indicate that depressed individuals are characterized by neural anomalies during the suppression of long-term memories, increasing our understanding of the brain bases of negative cognitive biases in MDD.

**Electronic supplementary material:**

The online version of this article (doi:10.3758/s13415-016-0464-x) contains supplementary material, which is available to authorized users.

Cognitive theories of major depressive disorder (MDD) suggest that negatively biased cognitive processes play a critical role in the development and maintenance of depression (e.g., Beck, 1976). Negative cognitions are posited to reflect dysfunctional schemas about the self that bias individuals’ processing toward negative-content stimuli. Indeed, depressed individuals exhibit enhanced memory for negative information (e.g., Mathews & MacLeod, 2005; Matt, Vázquez, & Campbell, 1992; Williams, Watts, MacLeod, & Mathews, 1997) and are characterized by deficits in controlling the processing of negative material (for a review, see Gotlib & Joormann, 2010). Theorists have suggested that these difficulties involving the processing of negative information contribute to the onset and severity of MDD (e.g., Ingram, 1984; Kircanski, Joormann, & Gotlib, 2012; Teasdale, 1983). Therefore, it is critical that we gain a better understanding of how depressed individuals process and control negative information.

One line of research, focusing on the functioning of working memory (WM), indicates that depressed individuals are characterized by deficits in inhibitory control (Gotlib & Joormann, 2010). For example, Joormann (2004) and Gotlib, Yue, and Joormann (2005) found that dysphoric individuals show reduced negative priming for negative stimuli; similar results were also reported by Goeleven, De Raedt, Baert, and Koster (2006) with depressed individuals. In these tasks, participants are asked to attend to, and make a judgment about, one stimulus while ignoring a second, simultaneously presented, stimulus. The negative-priming effect refers to a behavioral slowing that occurs when participants must respond to a stimulus that they were asked to ignore on the previous trial. This slowing is posited to reflect the lingering effects of inhibitory processes that were initiated when participants were required to ignore the stimulus when it was previously presented. Findings that depressed individuals show a reduced negative-priming effect for negatively valenced stimuli suggest that they have difficulty inhibiting the processing of negative items, and thus instead attend to the items they are supposed to ignore. Other studies have demonstrated that depressed individuals have difficulty *removing* no-longer-relevant negative information from WM (e.g., Joormann & Gotlib, 2008; Joormann, Nee, Berman, Jonides, & Gotlib, 2010; Levens & Gotlib, 2010). In these experiments, participants briefly memorize two sets of words and then are immediately instructed to ignore one of the sets to make a subsequent judgment about the words. Depressed individuals have difficulty when they are required to ignore sets of negative words (Joormann & Gotlib, 2008; Joormann et al., 2010), indicating impairment in removing negative information from WM.

Collectively, these studies indicate that depressed individuals are impaired in their ability to regulate the processing of negative stimuli. It is important to note, however, that these investigations have focused primarily on how depressed persons process novel or recently experienced information. It is likely that depressed individuals also struggle with inhibiting recall of long-term negative memories (Gotlib & Joormann, 2010). In fact, the accessibility of negative memories and the tendency to ruminate about them are hallmark characteristics of depression (Nolen-Hoeksema, 2000). One way in which researchers have studied individuals’ ability to inhibit unwanted long-term memories is by using the think/no-think (TNT) task (Anderson & Green, 2001). In this task, individuals learn paired associates (e.g., insect–roach) and are then asked to either practice retrieving (*think*) or suppressing the associates (*no-think*). The consequences of retrieving and suppressing are then assessed with a memory test in which all the learned associations are tested, including some pairs that the participants did not retrieve or suppress after the initial learning (i.e., baseline items). In nondepressed individuals, suppressing associates results in poorer subsequent memory for those words (Anderson & Green, 2001; for a review, see Anderson & Hanslmayr, 2014; Anderson & Huddleston, 2011). This finding of suppression-induced forgetting (SIF) suggests that attempting to prevent unwanted memories from entering awareness results in decreased long-term accessibility of those memories. With respect to depression, if depressed individuals have difficulty inhibiting retrieval, then they should show reduced SIF. Interestingly, the results of studies testing this formulation are mixed, with some showing that dysphoric and depressed individuals demonstrate less SIF than do healthy control participants (Hertel & Gerstle, 2003; Hertel & Mahan, 2008; Joormann, Hertel, Brozovich, & Gotlib, 2005), and others reporting no differences between dysphoric or depressed individuals and controls (Hertel & Calcaterra, 2005; Joormann, Hertel, LeMoult, & Gotlib, 2009). In fact, averaging across these five studies (weighted by sample size) yields data indicating that whereas control participants show a SIF effect of 5 % (effects that are consistent with, though slightly smaller than, those reported in other studies using unselected samples; e.g., Anderson & Huddleston, 2011), depressed participants show a SIF effect of 0 %, suggesting a modest deficit in inhibitory control in depression.^1^

The TNT paradigm has been adapted to study brain activity when individuals attempt to inhibit memory retrieval (Anderson, 2004; Benoit & Anderson, 2012; Butler & James, 2010; Depue, Curran, & Banich, 2007; Hulbert, Henson, & Anderson, 2016; Levy & Anderson, 2012; Paz-Alonso, Bunge, Anderson, & Ghetti, 2013). These studies have indicated that suppression is associated with increased activity in a set of regions including lateral prefrontal cortex (both dorsal and ventral regions), medial prefrontal cortex (including the anterior cingulate cortex and presupplementary motor area), and lateral parietal cortex (including the intraparietal sulcus and angular gyrus). This network is posited to be involved in the implementation of cognitive control that allows individuals to prevent unwanted memories from entering awareness. Increased activity in this network is accompanied by a decrease in medial temporal lobe activity during suppression, which is posited to be the target of top-down inhibitory regulation (Anderson, 2004; Depue et al., 2007; for a review, see Anderson & Hanslmayr, 2014). Consistent with this possibility, several studies have used effective connectivity analysis to document top-down modulation of hippocampal activity during retrieval suppression (Benoit & Anderson, 2012; Benoit, Hulbert, Huddleston, & Anderson, 2015; Gagnepain, Henson, & Anderson, 2014). Importantly, suppression is also associated with decreased activity in the amygdala when the items being suppressed are negatively valenced, suggesting a modulation of emotional response during these experiences (Depue et al., 2007). The patterns of brain activity during suppression exhibited by these unselected samples of individuals provide a baseline for understanding impaired memory suppression in MDD.

To date, investigators have not examined the neural bases of the deficits in SIF that have been reported in depressed individuals. In the present study, we assessed both the cognitive and the neural correlates of memory suppression in depressed individuals and healthy controls. Participants first learned paired associates, in which the cue word was always neutral but the response word was either negative or neutral in valence. Next, we scanned participants as they completed the TNT phase, which required them to either retrieve or suppress some of the word-pair associates. Immediately after this phase, we tested participants’ memory for all of the paired associates while they were still in the scanner. On the basis of previous findings, we hypothesized that depressed participants would exhibit less SIF than would healthy controls. We also predicted that depressed participants would have difficulty suppressing negative memories in particular, although it should be noted that prior studies using the TNT paradigm failed to confirm this prediction (Hertel & Gerstle, 2003; Joormann et al., 2005). Consistent with previous reports of reduced cognitive control ability in depressed individuals, we further predicted that depressed participants would demonstrate less activity than would healthy controls in the recruitment of prefrontal and parietal regions during suppression attempts. Finally, we predicted that, as compared to healthy controls, depressed participants would exhibit greater activation in the hippocampus and amygdala during no-think trials, particularly when negative memories were suppressed, reflecting diminished control over these memories for depressed individuals.

## Method

### Participants

Nineteen healthy control (CTL) individuals and 18 individuals diagnosed with MDD participated in the study. The data from three CTL and two MDD individuals were excluded due to problems with task presentation in the scanner (MDD *N* = 1, CTL *N* = 1) or scanner function (CTL *N* = 1), discomfort in the scanner (CTL *N* = 1), or an inability to learn the word pairs (MDD *N* = 1). After excluding these five participants, we were left with 16 MDD participants (nine females, seven males) and 16 CTL participants (eight females, eight males). The CTL participants had no history of psychiatric disorders and had never taken psychotropic medication. All participants were recruited through online postings, were between 18 and 56 years of age, had no history of brain injury and no substance/alcohol abuse in the last six months, and met the requirements for MRI scanning (e.g., had no metal implants). The MDD participants were not comorbid for bipolar I or II (mania), psychosis, or learning disabilities. The depressed participants also met the DSM-IV criteria for current MDD using the Structured Clinical Interview for DSM (SCID; First, Dibbon, Spitzer, & Williams, 2004). All participants also completed the Beck Depression Inventory (Beck, Rush, Shaw, & Emery, 1979). Participants were compensated for their time, and all gave informed consent. The study was in compliance with the ethical standards set forth by the American Psychiatric Association and was conducted with approval from the Stanford University institutional review board.

### Think/no-think task

#### Materials

The critical stimuli for this study were 24 sets of words; each set included four words. Each set was designed to have two possible cues (e.g., Trunk or Street), both of which were neutral, and two possible response words, one of which was negatively valenced (e.g., Corpse) and the other of which had a neutral valence (e.g., Violin). These words were selected from the Affective Norms for English Words (Bradley & Lang, 1999), allowing us to assess the valence (negative response words, *M* = 2.1, *SD* = 0.5; neutral response words, *M* = 5.5, *SD* = 0.6; neutral cue words, *M* = 5.1, *SD* = 0.6) and arousal (negative response words, *M* = 5.0, *SD* = 0.9; neutral response words, *M* = 4.0, *SD* = 1.0; neutral cue words, *M* = 3.4, *SD* = 0.7) ratings for each set of items.

Each set was designed so the cue words would act as effective retrieval cues for either response, so that the assignments of cues to responses could be counterbalanced across participants (i.e., one participant learned Trunk–Corpse and Street–Violin, and another participant learned Trunk–Violin and Street–Corpse). Similarly, the assignments of words pairs to conditions (baseline, think, and no-think) were also counterbalanced across participants. This meant that there were a total of six counterbalancing conditions for the items (three conditions and two cue-to-response mappings). Importantly, the cues were always neutral; therefore, the cue itself (Trunk or Street) did not provide any information about the valence of the response word. The independent probes were also designed to uniquely cue each response word separately (e.g., Anatomy–Co____ for Corpse; Lessons–Vi___ for Violin). Independent probes are used in these type of paradigms to rule out several noninhibitory explanations of forgetting (for more information, see Anderson & Spellman, 1995). These sets were divided into three groups of eight items that rotated through the experimental conditions (think, no-think, and baseline). An additional six word pairs (all neutral items) were used as fillers throughout the experiment; thus, each participant learned a total of 54 word pairs (six filler, eight think–negative, eight think–neutral, eight baseline-negative, eight baseline-neutral, eight no-think–negative, and eight no-think–neutral).

#### Procedure

The TNT procedure consisted of three separate phases: learning, TNT, and test. The learning phase was completed outside the scanner; the TNT phase and test phase were conducted while participants were inside the bore of the MRI scanner, though fMRI data were collected only during the TNT phase. This procedure was used to minimize forgetting on the final test that might be due to changes in physical and mental context associated with getting out of the scanner.

##### Learning phase

Participants learned the cue–associate word pairs through a drop-off study–test training procedure. On study trials, participants were presented with an intact word pair for 5 s and were encouraged to form an association between the items. On test trials, the cue word appeared and participants had up to 5 s to verbally report the associate. For feedback, the correct associate was presented for 2 s after every test trial. An experimenter recorded whether or not the response was correct. If no response was given or the response was incorrect, that cue word was presented again at the end of the list (i.e., items recalled correctly dropped out of the set). This was repeated for each list until every correct associate was provided once. To make this learning phase easier for participants, we divided the large number of word pairs into smaller lists and tested each word pair three times across the whole learning phase. More specifically, participants initially learned lists of six word pairs at a time (i.e., they studied six pairs, and then were immediately given drop-off testing on those six items). After the participants had learned three lists of six pairs, they were given an 18-item drop-off test covering all three lists they had just learned. Once this was complete, they moved on to three more lists of six items, followed by an 18-item drop-off test reviewing all of those pairs. After a third list of 18 items was learned, participants were given a drop-off cycle on all 54 word pairs they had learned. Once completed, participants were given one final test for all the cue–associate pairs to confirm which word pairs had actually been learned. During this last test, participants were not given feedback after making their response, and items they missed did not appear again at the end of the cycle. To minimize any potential differences in learning between the conditions (e.g., between negative and neutral items or between the MDD and CTL groups), all subsequent analyses were restricted to cue–associate pairs that were correctly reported on this final learning test. This allowed us to be sure that any differences we observed were not due to differences in initial learning.

##### TNT phase

For each trial, participants saw a cue from one of the word pairs (e.g., Street) and were asked to exert control over the retrieval process. For *think* trials, they were asked to recall the associated word (e.g., Corpse). For *no-think* trials, their task was to prevent the associated word from entering consciousness. Participants were not given any specific suggestions about strategies they could use to accomplish this task. Each retrieval cue was presented for 3 s, and they were asked to follow the task instructions for the entire time the cue was presented. Participants were cued to perform either of these tasks by the color of the cue word: Think cues appeared in green, and no-think cues were red. Participants completed a practice block that was 20 trials long and that included only filler pairs, to get the participants used to the procedure before scanning began. After this practice phase, they were asked about their approach to the task and given directed feedback if they were not performing the task as instructed (e.g., if they averted their gaze from the retrieval cue or covertly rehearsing the responses for no-think trials). The actual TNT phase consisted of six runs of 64 trials each (384 trials total); each run lasting 5 min 40 s. Each cue was repeated twice during every block (eight think and eight no-think cues of both valences, each presented twice). The trial order was determined by Optseq (http://surfer.nmr.mgh.harvard.edu/optseq; Dale, 1999), which pseudorandomly mixed the four conditions (think–negative, think–neutral, no-think–negative, and no-think–neutral) and used variable intertrial intervals (0.5–12 s). During the intertrial intervals a fixation cross appeared in the center of the screen, and participants were instructed to look at the cross and wait for the next trial to begin.

##### Test phase

**Fig. 1:**
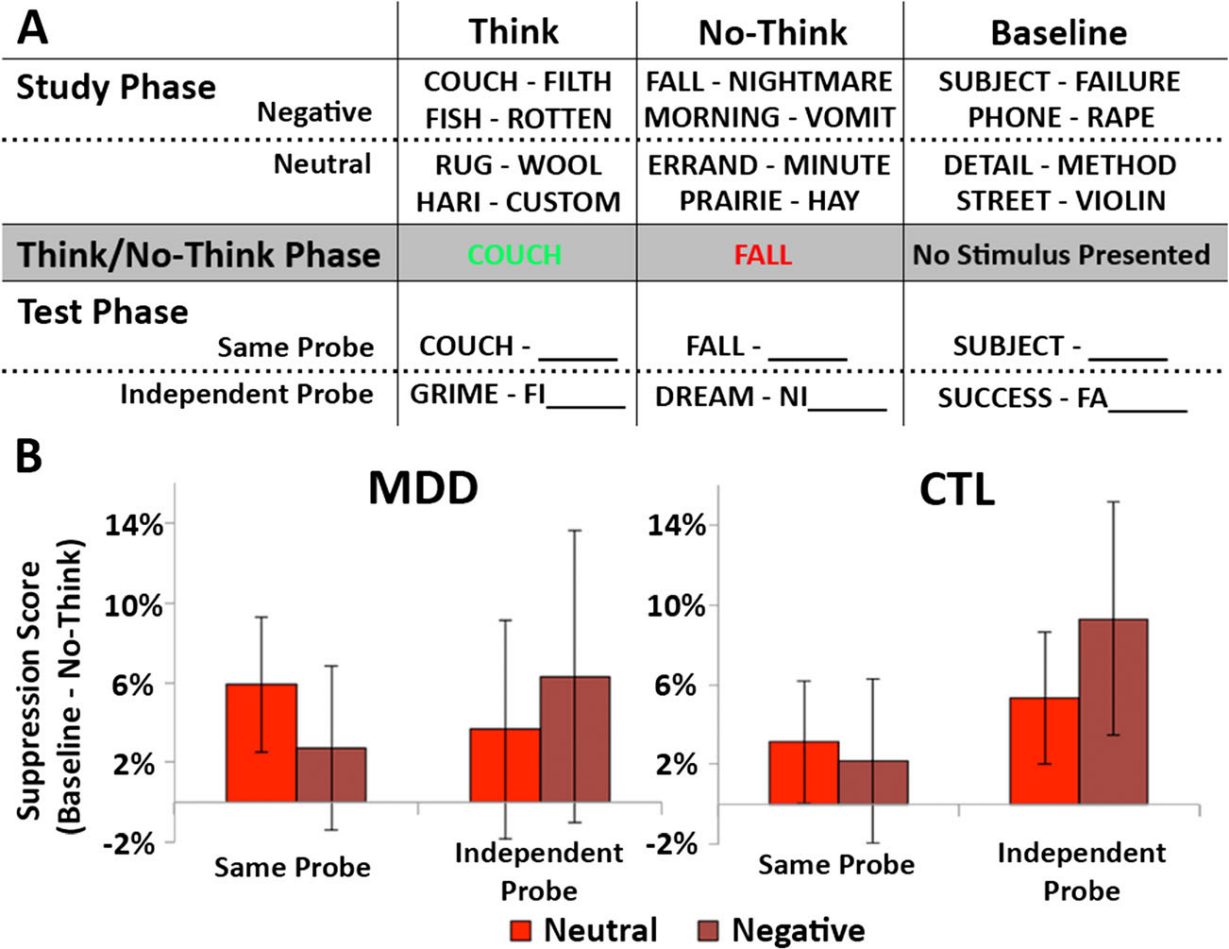
Behavioral procedure (A) and results (B). (A) During the study phase, participants learned word pairs until they could provide the associated member of each pair when shown the cue word as a retrieval cue. Then, during the think/no-think (TNT) phase, participants were scanned while they tried to exert control over memory retrieval. For think trials (in green during the trial), participants were asked to think of the associated word. For no-think trials (in red), they were asked to prevent the related word from entering awareness. Baseline items were not presented during this phase. After scanning, participants were asked to recall all of the studied response words, from both the originally studied retrieval cue (the same-probe test) and from a novel, extralist associate (the independent-probe test). (B) The critical outcome measure in the TNT task was the suppression score, which reflected whether or not avoided memories were recalled more poorly than baseline items (Baseline recall – No-think recall). Shown here are the suppression scores for both groups of participants (MDD and CTL), as a function of the valence of the to-be-suppressed memory (neutral or negative) and the type of final memory test (SP or IP). Overall, participants tended to forget the no-think items, and these suppression scores did not vary significantly by group, valence, or test type. The full set of means for recall in the final test phase are reported in Table 4. Error bars indicate standard errors of the means (*SEM*s).

After the end of MRI scanning, memory was tested for all word pairs. Participants were administered a brief practice test that tested only filler word pairs, to make sure that they understood the task. Then they were given two final memory tests, the *same-probe* (SP) and *independent-probe* (IP) tests, with the order of these two tests counterbalanced across participants. For each test trial, a retrieval cue was presented for 4 s, and participants were asked to verbally provide the associated word. The retrieval cue for the SP test was the cue from the originally studied word pair, and for the IP test it was a semantically related but unstudied cue along with a two-letter stem (Fig. 1A).

### Behavioral measures

The data from the test phase were analyzed to assess the behavioral consequences of attempting to control conscious awareness of a memory. A 2 × 2 × 2 × 3 mixed analysis of variance (ANOVA) was utilized, with the between-subjects factor Group (MDD and CTL) and the within-subjects factors Test Type (SP, IP), Valence (negative, neutral), and Condition (think, baseline, no-think). To focus more directly on the key behavioral measure (i.e., the magnitude of SIF), suppression scores were calculated by subtracting the recall of no-think items from the recall of baseline items, within a given valence and within each test type. This measure provides an index of how successful participants were at forgetting the avoided associates, controlling for general forgetting that would be expected on a delayed memory test. This measure treated SIF as a positive value, so participants who forgot more of the no-think items would show larger suppression scores.

### MRI data acquisition

Whole-brain imaging data was acquired via a 3.0-T General Electric Signa MR scanner (Milwaukee, Wisconsin) at the Richard M. Lucas Center for Imaging at Stanford University School of Medicine. After a scout scan used for slice prescription, high-order shimming was performed for whole-brain distortion estimation until diminished returns were produced. Blood-oxygenation-level-dependent (BOLD) functional data were acquired using an eight-channel, whole-head coil from 31 axial slices with a spiral in–out pulse sequence (Glover & Law, 2001; TR = 2,000 ms, TE 30 ms, flip angle = 80°, FOV = 22 cm, number of frames = 170, in-plane resolution = 3.44 mm^2^, through-plane resolution = 4 mm). To anatomically localize the functional activations, a high-resolution structural scan (spoiled gradient echo: 156 slices, in-plane resolution = 0.86 × 0.86 mm, through-plane resolution = 1 mm, TE = 3.4 ms, flip angle = 15°, FOV = 22 cm) was collected after the BOLD scanning runs.

### FMRI data processing and analysis

Data processing and analysis was conducted using the Analysis of Functional Neuroimages (AFNI) software suite (National Institutes of Health; http://afni.nimh.nih.gov/; Cox, 1996) and MATLAB (The MathWorks Inc., Natick, MA). The BOLD images were slice-time-corrected, followed by motion correction with a Fourier interpolation algorithm. Data were not corrected further if sudden movements were less than 1 mm. A despiking algorithm was used to correct for movements between 1 and 3 mm by replacing motion-influenced acquisitions with outlier insensitive estimates. Specifically, a given TR was defined as an outlier if its BOLD value was greater than a standard deviation threshold. Outlier values were then replaced with values from a polynomial fit across all TRs, excluding outliers. Spatial smoothing was conducted with a Gaussian kernel (full width at half maximum = 4 mm). The data were high-pass filtered at 1 cycle/min and converted to percent signal change. Finally, individual participant maps were converted to the Talairach common template space (Talairach & Tournoux, 1998), which allowed for between-group comparisons.

Processed time series data were then submitted to a general linear model (Friston, Holmes, Worsley, & Poline, 1995) that included regressors for condition (think and no-think) and valence (negative and neutral), residual motion, and first-, second-, and third-order polynomial trends. The regressors of interest were convolved with a gamma-variate function that modeled a canonical hemodynamic response before inclusion in the model (Cohen, 1997), and betas were estimated.

To assess consistency with previous fMRI investigations of the TNT procedure (e.g., Anderson, 2004), whole-brain maps were first computed using paired *t* tests on a voxel-wise basis, to create contrasts between think and no-think trials. This was done separately for each group (CTL and MDD) and both valences (neutral and negative). This allowed us to assess the extent to which the basic pattern of suppression-related activations would be observed in each of our contrasts (e.g., the MDD group suppressing negative items).

Next, we assessed whether activations differed between the CTL and MDD groups and between valences. To do this, we computed voxel-wise mixed ANOVAs with the between-subjects factor Group (MDD, CTL) and the within-subjects factors Condition (think, no-think) and Valence (negative, neutral). We then tested the significance of the two-way interactions between group and condition (within neutral trials) to identify any regions that were differentially active in the MDD versus CTL groups during no-think trials. Finally, to also consider valence, we looked for any regions that showed a three-way interaction of group, condition, and valence. This allowed us to identify regions that depressed and nondepressed participants might recruit differentially during the suppression of negative material.

All of these analyses were conducted at the whole-brain level; we had strong a priori interest, however, in the hippocampus and the amygdala. Thus, for these regions, we also conducted analyses with a small-volume correction (SVC), defined by probabilistic cytoarchitecture maps derived from postmortem brains (Eickhoff et al., 2005). For a given region, a voxel was included if at least 50 % of postmortem brains indicated that the voxel was identified as that region. Given that these were neighboring regions, a single search space was created for each hemisphere by combining hippocampal regions (cornu ammonis, entorhinal cortex, dentate gyrus, and subiculular complex) and amygdalar regions (centromedial, laterobasal, and superficial groups). The resulting bilateral hippocampus/amygdala volume was used for SVC, for a total volume of 23,960 mm^3^ (2,995 voxels). In the Results section, we report which clusters were identified in the whole-brain analysis, and which only survived using the SVC.

To control for multiple hypothesis testing while identifying significant outcomes, cluster-wise correction was implemented using 10,000 Monte Carlo simulations (Xiong, Gao, Lancaster, & Fox, 1995) using AFNI’s AlphaSim program. For whole-brain analyses, the uncorrected voxel significance threshold was set to *p* = *.*005, requiring a cluster of 256 mm^3^ (*k* = 32 voxels) to reach a corrected significance level of *p* < .05. To reach a corrected *p* < .05 significance level in the SVC analysis of the combined hippocampus/amygdala, the voxel-wise threshold was set to *p* < .05, and the required cluster sizes were 352 mm^3^ (*k* = 44 voxels) for left and 368 mm^3^ (*k* = 46 voxels) for right hippocampus/amygdala. Thus, both whole-brain and SVC analyses maintained a family-wise Type I error rate at *p* < .05. A more conservative voxel-wise *p*-value threshold was adopted for the whole-brain analysis, to reduce false positives, improve localization, and facilitate interpretation (Chrastil, Sherrill, Hasselmo, & Stern, 2015; Woo, Krishnan, & Wager, 2014).

To further explore significant the two- and three-way interactions involving group, time courses were extracted from significant clusters, and summary average signal estimates were computed across the second, third, and fourth time points (TRs 2–4, encompassing the expected activation peak), consistent with prior studies of the TNT paradigm (e.g., Levy & Anderson, 2012).

### Correlational analyses

Prior studies had found that behavioral measures of forgetting correlated with brain activity in the prefrontal cortex and within the medial temporal lobes (e.g., Anderson, 2004; Depue et al., 2007; Levy & Anderson, 2012). Similarly, studies had also reported correlations between the activity in different brain regions (e.g., Anderson, 2004; Benoit & Anderson, 2012; Depue et al., 2007), providing insights into how regions might interact during this task. Therefore, we attempted to replicate these analyses, but the analyses were inconclusive, so we report them only in the supplement (see the Supplemental Results). We also performed a series of exploratory analyses to explore whether activity in our regions of interest correlated with various clinical and psychological characteristics (see the Supplemental Method). Given the exploratory nature of these analyses, we also report the results of these analyses in the supplement (see the Supplemental Results).

## Results

### Participant characteristics

Demographic and clinical characteristics of the MDD and CTL participants are presented in Table 1. The depressed and nondepressed groups did not differ in age [*t*(30) = 0.06, *p* = *.*96], years of education [*t*(30) = 1.08, *p* = *.*29], or gender [*χ* ^2^(1, *N* = 32) = 0.13, *p* = *.*72]. As expected, MDD participants scored higher than did CTL participants on the BDI [*t*(30) = 12.0, *p* < .01]. Additional clinical characteristics for each of the MDD participants are presented in Table 2.

**Table 1:** Participant demographics

	MDD		CTL	
	*M*	*SD*	*M*	*SD*
Age (Years)	31.5	8.9	31.7	10.0
Education (Years)	14.5	2.5	15.4	2.5
BDI (Total score)	31.1	9.5	1.8	2.2
Female	56.25 %		50 %	

MDD = depressed participants; CTL = control participants; BDI = Beck Depression Inventory I or II

**Table 2:** MDD participant clinical data

Participant	Comorbidity	Medication (Daily Dosage [mg], Duration [Months])	MDD-Related Hospitalizations	Current Episode Duration (Months)	Years Since First Episode	BDI Score
1	None	Buprenorphine (4, 8); Zolpidem (10, 11)	0	5	17	30
2	None	None	0	9	30	29
3	DYS, anxiety disorder NOS	Lorazepam (0.5, 120)	1	8	45	22
4	Social phobia	None	0	N.R.	30	16
5	None	None	0	3	1	38
6	None	Lorazepam (2, 18)	0	144	15	29
7	None	None	0	2	11	36
8	None	Lithium (NR, 24); Fluvoxamine (300, 24)	1	120	10	24
9	None	None	1	1	14	36
10	None	None	0	1	23	31
11	GAD	None	1	3	20	21
12	None	Sertraline (100, 24)	0	1	21	26
13	None	None	0	3	23	54
14	None	Bupropion (300, 12)	0	3	4	27
15	DYS	None	0	2	10	32
16	None	Lithium (600, 18)	0	192	16	46

MDD = major depressive disorder; DYS = dysthymia; GAD = generalized anxiety disorder; N.R. = not reported; BDI = Beck Depression Inventory I or II

### Learning performance

The drop-off learning procedure ensured that both groups of participants had learned most of the word pairs by the time they got to the last practice test (see Table 3). The CTL group recalled nonsignificantly more words (93.9 %) than did the MDD group (89.7 %) [*t*(30) = 1.12, *p* = *.*28]. Similarly, the interaction between group and valence was not significant [*F*(1, 30) = 2.07, *p* = *.*16]. All subsequent analyses were conditionalized on learning so as to ensure that the results were not influenced by learning differences (i.e., the behavioral and neuroimaging analyses only considered data from word pairs that were correctly recalled during this final practice test).

**Table 3:** Performance on the final learning test before the TNT phase

	MDD		CTL	
	*M*	*SD*	*M*	*SD*
All associates	89.7 %	11.2 %	93.9 %	8.3 %
Negative associates	87.8 %	14.0 %	93.5 %	9.0 %
Neutral associates	91.7 %	9.3 %	94.3 %	8.0 %

MDD = depressed participants; CTL = control participants

### Final recall performance

To assess the behavioral consequences of exerting control over retrieval, we investigated recall performance on the final memory test as a function of group (MDD and CTL), condition (think, baseline, and no-think), valence (negative and neutral), and test type (SP and IP; see Table 4 for the full set of means). This analysis yielded a main effect of test type [*F*(1, 30) = 161.0, *p* < .001], reflecting better recall on the SP test (90.9 %) than on the IP test (58.8 %). This effect is not surprising, given that novel semantic associates typically produce poorer recall than do retrieval cues that were present at encoding (e.g., Tulving & Thomson, 1973). The analysis also yielded a main effect of condition [*F*(2, 60) = 3.77, *p* < .05], which reflects the fact that no-think items (71.9 %) were recalled less frequently than baseline items (76.7 %) [*F*(1, 30) = 9.88, *p* < .005], replicating the basic SIF effect (see Anderson & Huddleston, 2011). No main effects were obtained for group or valence (*F*s < 1), and none of the possible interactions were significant.

**Table 4:** Recall performance on the final memory tests (conditionalized on correct initial learning)

			MDD		CTL	
			*M*	*SD*	*M*	*SD*
Same probe	Think	Neg	89.6 %	11.8 %	93.8 %	15.3 %
		Neu	94.6 %	11.6 %	97.7 %	5.0 %
	No-think	Neg	85.6 %	16.3 %	91.1 %	12.4 %
		Neu	84.7 %	18.4 %	89.4 %	12.4 %
	Baseline	Neg	88.3 %	15.2 %	93.3 %	10.6 %
		Neu	90.6 %	13.7 %	92.5 %	8.2 %
Independent probe	Think	Neg	58.6 %	20.3 %	56.9 %	26.4 %
		Neu	61.7 %	17.2 %	54.7 %	27.9 %
	No-think	Neg	59.7 %	18.2 %	56.4 %	22.2 %
		Neu	53.2 %	18.7 %	55.3 %	22.1 %
	Baseline	Neg	66.0 %	19.7 %	65.7 %	23.1 %
		Neu	56.8 %	16.5 %	60.6 %	20.5 %

MDD = depressed participants; CTL = control participants; Neg = negatively valenced words; Neu = neutral words

Although the absence of any significant interactions suggests that SIF was not modulated by group or valence, a limitation of these analyses is that they also included think performance in the condition variable. Our a priori interest was specifically in differences in SIF, which is defined as the difference in recall between the no-think and baseline conditions. Therefore, we also conducted planned comparisons examining the difference between no-think and baseline suppression scores (see Fig. 1B), as a function of group and valence. Contrary to our predictions, the magnitude of these suppression scores did not interact with any combination of valence, group, or test type (*F*s < 1), consistent with the pattern observed in the overall ANOVA. Thus, we found no evidence of any differences in SIF as a function of the valence of the words or depression group status.

### FMRI results

**Fig. 2:**
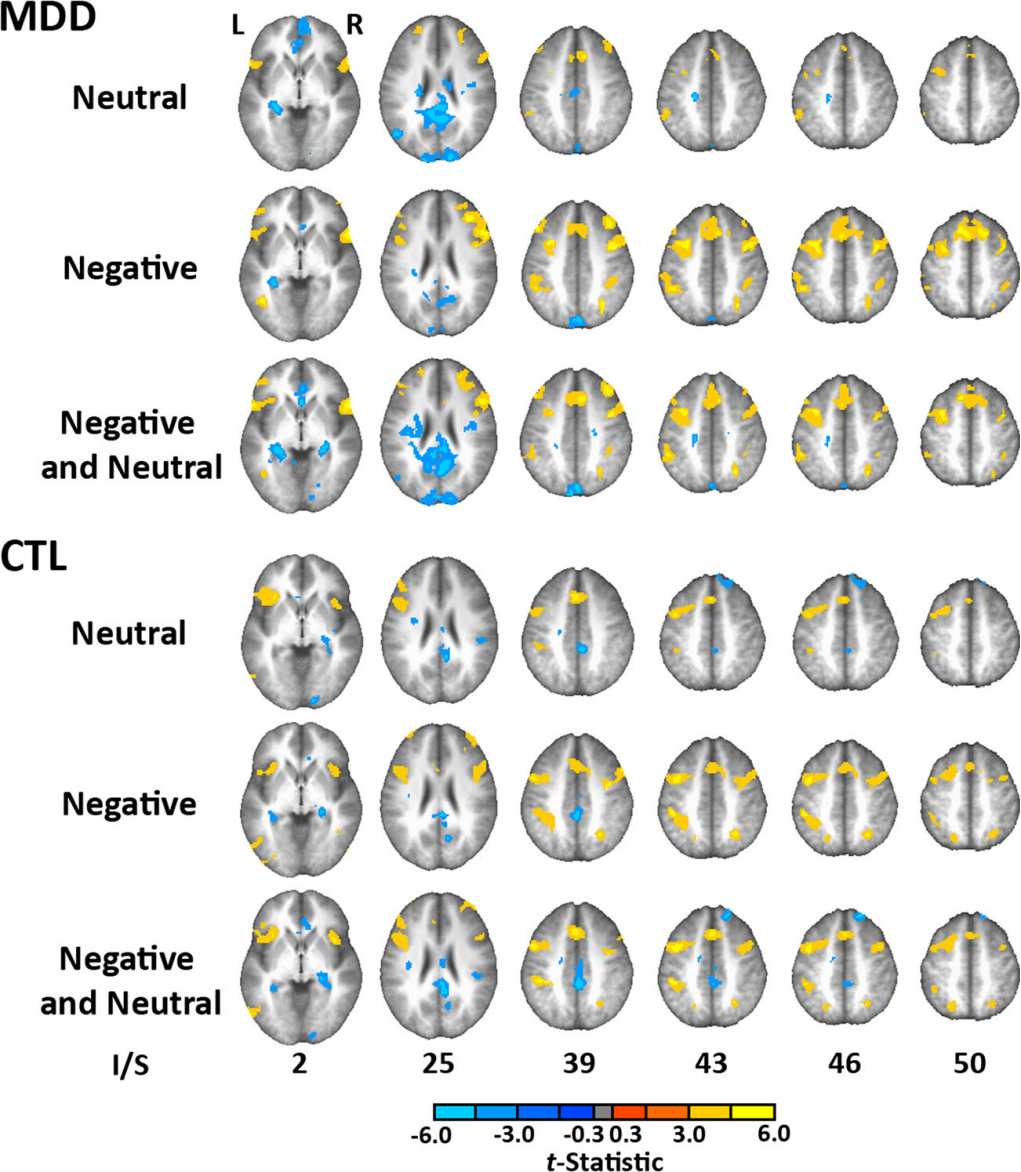
Comparison of brain activity during think and no-think trials. Warm colors indicate increased activity during no-think as compared to think trials, and cool colors indicate decreased activity during no-think as compared to think trials. The top three rows depict activity for the MDD group, and the bottom three rows display data from the CTL group. Each row represents the valence of the to-be- suppressed item (neutral, negative, or collapsing across both valence types). In general, the patterns for both groups and across all combinations of valence resemble prior findings using this paradigm: No-think trials are associated with increased activity in prefrontal and parietal regions and decreased activity in the medial temporal lobes and in medial parietal regions. L = left, R = right

For the imaging results, we began by comparing the brain activity during think and no-think trials, separately for each group and each valence (see Table 5 and Fig. 2). We did this, in part, to facilitate comparisons with previous imaging studies that had used the TNT task (e.g., Anderson, 2004; Benoit et al., 2015; Butler & James, 2010; Depue et al., 2007; Gagnepain et al., 2014; Levy & Anderson, 2012). First, we examined CTL participants and neutral stimuli, because this condition was most comparable to those in the majority of prior studies. Importantly, we replicated the typical pattern of activity: increased activity during no-think trials in prefrontal (including inferior and middle frontal gyrus, premotor, and supplementary motor cortex) and lateral parietal cortex, along with decreased activity during no-think trials in the medial temporal lobes and medial parietal cortex. Similarly, when CTL participants engaged with negative stimuli, a condition that was explored in two prior studies (Butler & James, 2010; Depue et al., 2007), we again replicated the patterns described above. The novelty of the present study, of course, was the inclusion of MDD participants engaged in the same task as CTL participants. Here we observed a similar pattern to that in the CTL group when depressed individuals exerted control over either neutral or negative stimuli, with all of the key patterns being replicated in these contrasts. This suggests that the basic regions involved in memory suppression are broadly similar across depression statuses and valences. This analysis was not sufficient, however, to assess whether any group- and valence-related differences would emerge in the neural aspects of memory suppression. Therefore, we conducted analyses focused on assessing whether these other factors (Group and Valence) influence brain activity during the TNT task.

**Table 5:** Differences in brain activity between think and no-think trials, separated by group (MDD and CTL) and valence (neutral and negative)

Laterality	Region	Max *t* Value	Tal L/R, A/P, I/S (mm)	Cluster Extent (voxels, 1 voxel = 8 mm^3^)
MDD Group, Neutral Items				
*No-Think > Think*				
R	Inferior frontal gyrus	5.431	+53.0 +17.0 +4.0	567
R	Premotor	5.861	+5.0 +11.0 +58.0	503
R	Middle frontal gyrus	6.237	+35.0 +33.0 +34.0	492
R	Cingulate	5.586	+7.0 +17.0 +34.0	277
L	Inferior frontal gyrus	4.955	–53.0 +15.0 +6.0	244
L	Middle frontal gyrus/inferior frontal gyrus	4.537	–43.0 +27.0 +32.0	125
L	Inferior parietal	4.541	–49.0 –41.0 +44.0	117
L	Middle frontal gyrus/frontal pole	4.216	–21.0 +47.0 +26.0	76
L	Interior temporal pole	5.672	–47.0 –9.0 –34.0	54
L	Orbitofrontal	4.412	–17.0 +57.0 –14.0	39
*Think > No-Think*				
L	Posterior cingulate	–7.425	–11.0 –37.0 +32.0	2,201
L	Parieto-occipital	–5.161	–5.0 –85.0 +38.0	796
L	Medial prefrontal	–5.479	–5.0 +27.0 +4.0	418
L	Hipppocampus	–5.931	–23.0 –33.0 +4.0	266
L	Parieto-occipital	–5.447	–45.0 –65.0 +24.0	177
L	White matter	–6.709	–19.0 –21.0 +22.0	133
R	White matter	–5.345	+13.0 –13.0 +28.0	82
R	White matter	–4.323	+19.0 –21.0 +22.0	75
L	White matter	–4.186	–31.0 –57.0 +8.0	63
L	White matter	–5.497	–17.0 –25.0 +44.0	38
R	White matter	–3.968	+31.0 –15.0 +28.0	32
CTL Group, Neutral Items				
*No-Think > Think*				
L	Inferior frontal gyrus	5.286	–43.0 +37.0 +22.0	793
L	Inferior frontal gyrus and middle frontal gyrus	5.087	–39.0 –1.0 +46.0	735
L	Supplementary motor area	5.694	–3.0 +19.0 +42.0	397
L	Inferior temporal	5.151	–57.0 –57.0 –6.0	228
R	Insula	4.448	+5.0 +13.0 +2.0	112
R	Premotor	5.086	+13.0 +9.0 +60.0	99
L	Inferior parietal	4.120	–41.0 –35.0 +42.0	62
R	Temporal pole	4.801	+45.0 +9.0 –16.0	60
L	Premotor	3.985	–1.0 +9.0 +60.0	57
L	Cerebellum	4.149	–33.0 –51.0 –26.0	39
*Think > No-Think*				
R	Posterior cingulate cortex	–5.715	+5.0 –35.0 +38.0	568
L	White matter	–4.515	–25.0 –9.0 +30.0	138
R	White matter/posterior medial temporal lobe	–4.370	+17.0 –23.0 +6.0	120
R	Occipital lobe	–5.127	+13.0 –89.0 +0.0	108
R	Superior frontal gyrus	–4.575	+13.0 +33.0 +44.0	93
R	Ventral anterior cingulate	–6.545	+1.0 +25.0 –6.0	77
R	Cingulate gyrus	–3.936	+5.0 –13.0 +34.0	73
R	Frontal pole	–4.225	+3.0 +61.0 +14.0	69
L	Cerebellum	–4.376	–31.0 –69.0 –28.0	68
R	Inferior parietal	–3.775	+43.0 –29.0 +24.0	60
MDD Group, Negative Items				
*No-Think > Think*				
R	Frontal	7.062	+33.0 +31.0 +28.0	2,293
R	Premotor	7.183	+7.0 +13.0 +54.0	1,596
L	Premotor	6.233	–27.0 +7.0 +42.0	602
L	Inferior parietal	5.426	–45.0 –45.0 +44.0	373
L	Middle frontal gyrus	7.141	–45.0 +23.0 +36.0	350
R	Inferior parietal	4.723	+37.0 –35.0 +38.0	280
L	Inferior temporal gyrus	6.470	–57.0 –43.0 –10.0	253
L	Inferior frontal gyrus	4.701	–49.0 +17.0 +4.0	245
R	Inferior parietal sulcus	5.753	+25.0 –61.0 +42.0	137
L	Inferior frontal gyrus	4.285	–37.0 +49.0 –6.0	122
L	Inferior frontal gyrus/middle frontal gyrus	4.638	–43.0 +7.0 +28.0	100
L	Inferior temporal gyrus	4.378	–39.0 –53.0 –12.0	84
R	White matter	4.021	+17.0 –1.0 +10.0	66
L	Orbitofrontal	5.357	–11.0 +19.0 –20.0	48
R	Orbitofrontal	5.116	+31.0 +47.0 –8.0	43
R	Middle temporal gyrus	4.586	+47.0 –33.0 –4.0	42
R	Frontal temporal	4.405	+29.0 +13.0 –18.0	34
*Think > No-Think*				
R	Parietal–occipital	–6.395	+3.0 –79.0 +38.0	528
R	Precuneus	–5.223	+15.0 –55.0 +24.0	296
L	Precuneus	–4.217	–13.0 –55.0 +18.0	195
L	Hippocampus	–5.608	–29.0 –37.0 +2.0	155
R	Brainstem	–4.446	+5.0 –35.0 –38.0	50
R	White matter	–4.448	+1.0 +21.0 +4.0	47
L	White matter	–4.316	–27.0 –29.0 +24.0	42
L	Cingulate	–3.775	–7.0 –41.0 +34.0	40
R	Cerebellum	–4.987	+21.0 –43.0 –34.0	36
R	White matter	–4.252	+17.0 –37.0 +6.0	32
CTL Group, Negative Items				
*No-Think > Think*				
L	Frontal	5.428	–39.0 +1.0 +44.0	1,209
R	Middle frontal gyrus/superior frontal gyrus	6.080	+43.0 –1.0 +36.0	817
R	Cingulate	4.509	+13.0 +11.0 +38.0	559
L	Inferior temporal	4.953	–47.0 –55.0 –4.0	495
L	Inferior Parietal	5.432	–43.0 –35.0 +44.0	420
L	Inferior frontal gyrus/middle frontal gyrus	5.762	–29.0 +35.0 +14.0	365
R	Inferior frontal gyrus	4.875	+39.0 +25.0 +10.0	300
L	Occipital	5.662	–35.0 –85.0 +10.0	206
R	Inferior parietal sulcus	5.711	+25.0 –57.0 +44.0	193
R	Middle frontal gyrus	4.235	+37.0 +47.0 +26.0	151
L	Superior parietal	5.091	–17.0 –67.0 +54.0	98
R	Inferior temporal	4.078	+49.0 –71.0 +0.0	79
R	Occipital	4.845	+35.0 –83.0 +16.0	65
R	Cerebellum	3.894	+33.0 –59.0 –10.0	38
*Think > No-Think*				
L	Cingulate	–5.841	–1.0 –37.0 +38.0	457
L	White matter	–5.293	–33.0 –31.0 +4.0	157
R	Precuneus	–5.519	+9.0 –59.0 +22.0	152
R	Hippocampus	–4.923	+21.0 –29.0 +2.0	145
R	Middle temporal gyrus	–5.633	+55.0 –15.0 –8.0	122
R	Brainstem	–5.567	+3.0 –39.0 –32.0	83
R	Cingulate	–4.369	+5.0 –17.0 +32.0	73
L	Insula	–4.380	–39.0 –13.0 +12.0	71
R	White matter	–4.789	+9.0 +27.0 +0.0	55
R	Middle temporal	–3.998	+47.0 +3.0 –18.0	40

**Fig. 3:**
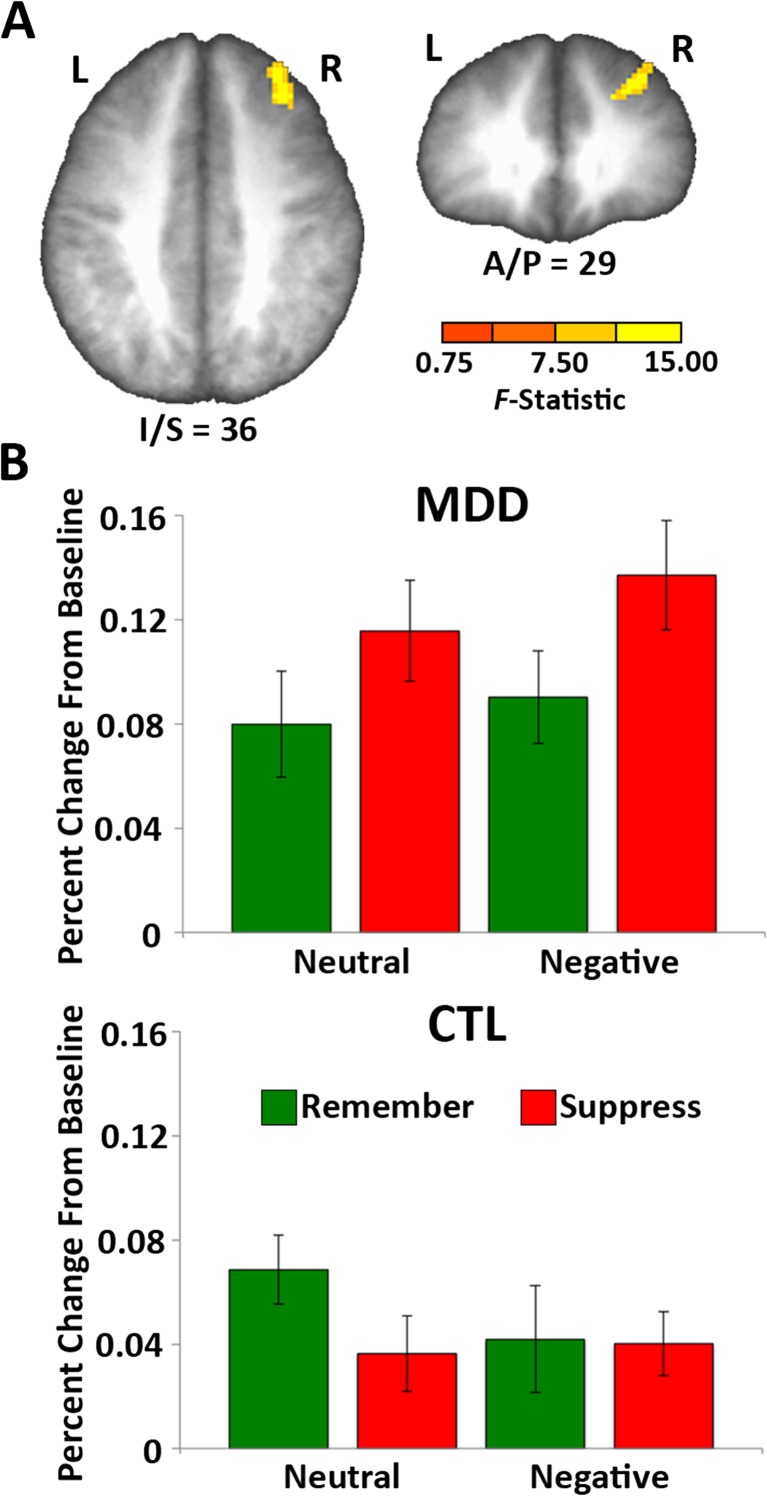
The MDD group, but the not CTL group, activated the right middle frontal gyrus (MFG) during memory suppression. (A) This cluster (Talairach coordinates: 29 [R], 29 [A], 38 [S]; 1,136 mm^3^, 142 voxels) was identified by a whole-brain analysis looking for regions that showed a group (MDD or CTL) by condition (think or no-think) interaction (limited to neutral trials). (B) The percent signal change was then extracted from this cluster for each condition to further examine this interaction. This revealed that the MDD group recruited this region more during no-think than during think trials, whereas control participants did not. This pattern held for both neutral trials, which had been used to identify the cluster, and for an independent (noncircular) analysis of the negative trials. Error bars indicate standard errors of the means (*SEM*s). L = left, R = right

To assess the differences between depressed and nondepressed individuals, we first looked for regions that showed a two-way interaction of condition (think, no-think) and depression status (MDD, CTL). We restricted this analysis to neutral items so we could focus on whether depressed and nondepressed individuals differed in how they approached the task, without considering the negative stimuli that we expected would be particularly challenging for depressed individuals. Although we found no differences in the behavioral consequences of suppression for the two groups of participants, depressed individuals might nevertheless have achieved the same outcome by recruiting different neural processes than those recruited by their nondepressed counterparts. We obtained a significant interaction of group and condition in three clusters (Table 6), two of which were located in the right middle frontal gyrus (MFG), a region we hypothesized might be engaged differentially by depressed and nondepressed individuals during suppression. To better understand this interaction, we extracted the time courses from the larger MFG cluster (Fig. 3). Looking at neutral trials, the interaction is driven by the MDD group recruiting this region to a greater extent during no-think than during think trials [*F*(1, 30) = 9.25, *p* < .005], whereas the CTL group showed the opposite pattern [*F*(1, 30) = 7.49, *p* < .05]. Interestingly, although this region was identified on the basis of neutral trials alone, a similar pattern was observed in this region during negative trials. Specifically, the MDD group recruited the region more for no-think than for think trials [*F*(1, 30) = 6.80, *p* < .05], but the CTL group did not (*F* < 1). This interaction approached significance [*F*(1, 30) = 3.66, *p* = *.*06], providing weak support for the formulation that the right MFG is recruited during suppression more by depressed than by nondepressed participants, and it does so in an independent (i.e., noncircular) comparison.

**Table 6:** Brain regions identified by the two-way interaction of condition (Think and No-Think) and group (MDD and CTL) for neutral items only

Laterality	Region	Max *F* Stat	Tal L/R, A/P, I/S (mm)	Cluster Extent (voxels, 1 voxel = 8 mm^3^)
R	MFG	13.772	29, 29, 38	137
L	Ventral striatum/white matter	29.694	–13, –1, –6	53
R	MFG/SFG	17.564	21, 55, 30	37

**Fig. 4:**
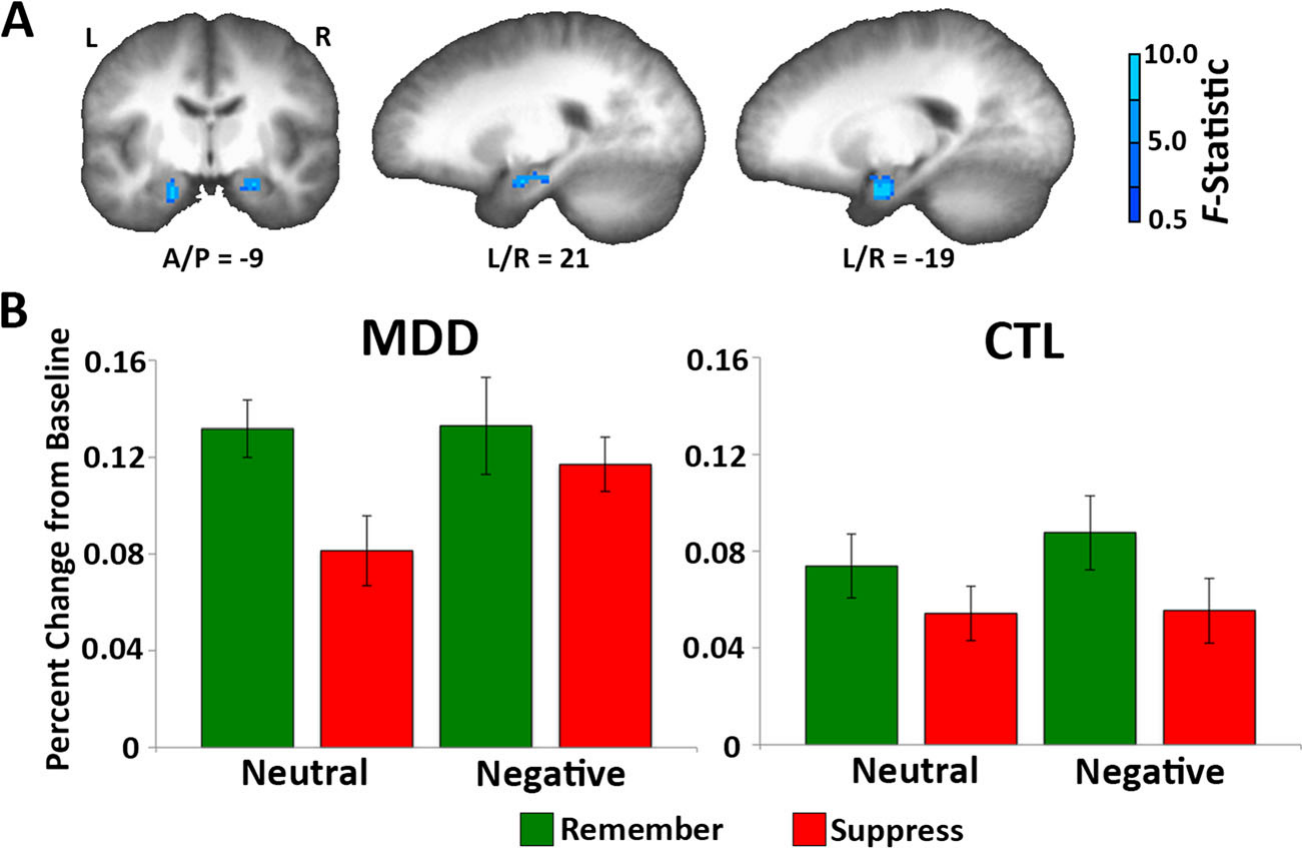
Modulation of the targets of memory control differs across depression statuses and valences. (A) Using a small-volume correction, a three-way interaction between group, valence, and condition was identified in clusters that encompassed the amygdala and anterior hippocampus in both the right hemisphere (Talairach coordinates: 21 [R], –9 [P], –14 [I]; 824 mm^3^, 103 voxels) and the left hemisphere (Talairach coordinates: –19 [L], –5 [P], –18 [I]; 704 mm^3^, 88 voxels). (B) The percent signal change was then extracted from a combined bilateral hippocampal–amygdala cluster for each condition, to further examine this interaction. This revealed that the MDD group showed the typical suppression-related down-regulation of this region during neutral trials, but not during negative trials. The CTL group showed the opposite pattern, with successful down-regulation for negative items. Error bars indicate standard errors of the means (*SEM*s). L = left, R = right

Next we examined whether the depressed and nondepressed participants differed in suppression-related activity when the valence of the to-be-suppressed item was manipulated. To do this, we looked for any region that showed a three-way interaction of group, condition, and valence. No regions emerged in this analysis at the whole-brain level; however, given our a priori interest in the hippocampus and amygdala, we conducted the same analysis within a small-volume-corrected search space based on probabilistic atlases of these areas. This analysis revealed clusters in both the right and left hemispheres (Fig. 4A) that spanned both the amygdala and the anterior portion of the hippocampus. To further investigate this three-way interaction, we extracted the time courses from a mask combining the two clusters (the locations were similar in both hemispheres, as was the ordering of the conditions). This analysis revealed that the MDD group modulated activity in this region, as evidenced by decreased activity during no-think relative to think trials, significantly for neutral items [*F*(1, 30) = 10.69, *p* < .005], but not for negative items (*F* < 1). Control participants showed significant modulation for negative items [*F*(1, 30) = 4.06, *p* = *.*05], but not for neutral items [*F*(1, 30) = 1.62, *p* = .21]. Importantly, however, the two-way interaction between condition and valence within each group was not significant for either MDDs [*F*(1, 30) = 1.94, *p* = .17] or control participants (*F* < 1).

## Discussion

The present experiment was the first fMRI study to investigate whether and how depressed individuals differ from their nondepressed counterparts in suppressing unwanted memories. Although we did not observe the expected behavioral impairment in memory suppression for MDD participants, we did find indications in the neuroimaging data concerning how they achieved memorial control. First, depressed individuals were more likely than controls to recruit the right MFG when required to suppress a memory. Second, when we considered the valence of the to-be-suppressed information, the MDD and CTL groups differed in how they modulated activity in the targets of memorial control, specifically the amygdala and hippocampus. These findings point to potential differences between depressed and nondepressed individuals that expand on prior work documenting deficits in inhibitory control in depressed individuals engaged in WM tasks (see Gotlib & Joormann, 2010).

Researchers have consistently reported that lateral prefrontal cortex (PFC) is engaged during memory suppression (Anderson, 2004; Benoit & Anderson, 2012; Benoit et al., 2015; Butler & James, 2010; Depue et al., 2007; Gagnepain et al., 2014; Levy & Anderson, 2012). This activation often spans both the ventral and dorsal aspects: the inferior frontal gyrus and MFG, respectively. Investigators have generally interpreted these lateral PFC activations as reflecting control processes that are engaged to prevent the unwanted memory from entering awareness (see, e.g., Anderson & Hanslmayr, 2014; Anderson & Levy, 2009). Although we found evidence broadly consistent with this general pattern in both the depressed and nondepressed groups, we observed engagement of a specific region within the right MFG that was specific to depressed individuals. Interestingly, this region appears to be anterior and superior to the right-MFG activations that are often seen in univariate contrasts in nondepressed participants. One possibility is that this is a distinct region that is recruited by depressed participants to suppress unwanted memories. Another possibility, which is not mutually exclusive of the first, is that the difference could reflect differential use of suppression strategies by the two groups. Participants in the TNT task report using a wide range of strategies (e.g., thinking of diversionary thoughts or attempting to let their mind go blank; see Levy & Anderson, 2008), and when participants are instructed to use different strategies, we observe differences in the recruitment of specific prefrontal regions (Benoit & Anderson, 2012). Benoit and Anderson found that participants selectively recruited a region near this area when they engaged in a direct suppression strategy in which they attempted to stop retrieval without generating alternative thoughts. Because in this study we did not instruct participants to use any specific strategy or ask them to report which strategies they used, we do not know whether the two groups used similar strategies to complete the task. One surprising aspect of this finding is that it reflects increased activity in MDD participants, whereas we predicted that PFC should be hypoactive for these individuals. In retrospect, however, we note that there have been widespread observations of heightened PFC recruitment in depression (e.g., Bär et al., 2007; Diener et al., 2012; Grimm et al., 2008; Harvey et al., 2005; Wagner et al., 2006; Walter, Wolf, Spitzer, & Vasic, 2007). Interestingly, Harvey et al. 2005 suggested that this hyperactivity is most likely to be observed when behavioral performance is matched to healthy controls, as was the case here, suggesting that this is a form of compensatory recruitment (e.g., perhaps due to strategy differences, as suggested above). Further research will be needed to replicate this unexpected pattern and to gain greater leverage on what this region contributes during the task.

In addition to increased PFC activity during suppression, it is also typical to observe decreased activity in the medial temporal lobes (Anderson, 2004; Benoit & Anderson, 2012; Benoit et al., 2015; Depue et al., 2007; Gagnepain et al., 2014; Levy & Anderson, 2012). This finding is often interpreted as indicating that the medial temporal lobes are a target of frontal cognitive control, and that their activation is down-regulated, which results in an override of their role in recovering information from long-term memory (e.g., Anderson & Hanslmayr, 2014; Anderson & Levy, 2009). In addition, there is evidence that when participants attempt to suppress negatively valenced information, they also down-regulate activity in the amygdala (Butler & James, 2010; Depue et al., 2007). In the present study, we observed a cluster in both hemispheres that included the amygdala as well as the hippocampus and that activated differentially in the depressed and nondepressed groups to the different valences. Across all combinations of valence and group, think activity in this region was numerically greater than no-think activity, consistent with prior evidence that these regions are modulated during suppression. The three-way interaction appears to reflect the differential effectiveness of this modulation between the groups for the two different valence conditions: Specifically, nondepressed participants were relatively more successful at modulating this region when suppressing negative materials, and depressed participants were relatively more successful when suppressing neutral stimuli. We expected that depressed individuals would have the most difficulty suppressing memories of negative experiences, so this pattern is broadly consistent with our predictions.^2^

It is interesting to note that we did not observe any significant behavioral differences between the depressed and nondepressed participants. This may not be surprising, given that other studies have also failed to observe significant SIF impairments in depressed and dysphoric populations (Hertel & Calcaterra, 2005; Joormann et al., 2009). Interestingly, the studies that have reported no significant differences in SIF as a function of depression status have tended to have smaller sample sizes than the studies that have reported significant differences, a concern that is also relevant to the present study. Interestingly, we did observe numerically greater SIF in CTLs than in MDDs on the IP test (7.3 % vs. 5.0 %, respectively, collapsed across valences), which has been argued to be more sensitive than the SP test for assessing inhibitory control ability (Anderson & Levy, 2011). When considering all of the behavioral evidence, it appears that the inhibitory impairments in this paradigm may be relatively modest. Similarly, despite our emphasis on the regions that differed between controls and depressed individuals, there was considerable similarity in the general patterns of brain activity observed in these two groups of participants, which is again consistent with the position that differences on this task due to suppression are subtle.^3^ That does not mean, however, that any differences would be unimportant for increasing our understanding of depression. Indeed, even small impairments in the moment-to-moment regulation of retrieval could compound dramatically, given the many retrieval opportunities that are present in everyday life. For example, we know that retrieval itself acts as a powerful learning event (see, e.g., Karpicke, 2012); thus, each instance of failed retrieval suppression could lead to that unwanted thought being encoded better in long-term memory and, in MDDs, contributing to negative schemas that have been posited to perpetuate the negative cognitions that are fundamental to this disorder (Beck, 1976). It is also important to note that, consistent with prior studies using this paradigm (Hertel & Gerstle 2003; Joormann et al., 2005), we did not observe any evidence of diminished forgetting of negative material by the depressed individuals. Although individual null results can be inconclusive, the repeated failure to confirm this prediction seems to be at odds with other evidence suggesting that depressed individuals have particular difficulty suppressing negative stimuli (for a review, see Gotlib & Joormann, 2010). Interestingly, all of the extant TNT studies have used verbal stimuli, which may not produce as strong an affective modulation as contextually richer stimuli (e.g., photographs). Perhaps future studies could use valenced picture stimuli (see Depue, Banich, & Curran, 2006; Depue et al., 2007) with these populations to further explore this issue.

In closing, our findings suggest that depressed and nondepressed individuals differ in their brain activity when they are asked to suppress memory retrieval. A fruitful next step would be to further explore the regions implicated in this study, to elucidate their mechanistic contributions to this task. This area of inquiry could also benefit from a behavioral study with a larger sample, in order to better characterize the magnitude and consistency of depression-associated behavioral impairments in this task. It would also be instructive to examine the brain activity as depressed individuals are provided with an explicit strategy to perform the suppression task. Previous research has suggested that “aided” strategies, in which an alternative diversionary thought is used, can help depressed individuals overcome deficits in SIF (Hertel & Calcaterra, 2005; Joormann et al., 2009), and can also change the pattern of brain activity in nondepressed individuals (Benoit & Anderson, 2012; Bergström, de Fockert, & Richardson-Klavehn, 2009). Finally, this line of research could help bridge two features that are often discussed as distinct aspects of depression: impaired inhibitory control and rumination. As we discussed earlier, most of the evidence for impaired inhibition in depression has focused on how items enter or are removed from WM. This has obvious implications for how individuals might encode new experiences, but it tells us relatively little about how they deal with existing long-term memories of their own experiences, which are precisely the kinds of events about which depressed individuals are likely to ruminate. Indeed, in an unselected sample of undergraduate students, levels of rumination were found to be related to impaired suppression-induced forgetting, even when controlling for deliberate reprocessing, which is related to both rumination and suppression (Fawcett et al., 2015; Joormann & Tran, 2009). Furthermore, Hertel and Gerstle (2003) found that dysphoric individuals who were high in rumination exhibited reduced SIF as compared to lower-ruminating dysphoric individuals. These findings implicate rumination as a key component of SIF abnormalities in MDD. Gaining a better understanding of how depressed individuals struggle to control access to memory will not only help elucidate the core features of depression, but may be relevant for other disorders that also involve difficulty controlling unwanted thoughts (e.g., Brewin, Gregory, Lipton, & Burgess, 2010; Catarino, Kupper, Werner-Seidler, Dalgleish, & Anderson, 2015; Depue, Burgess, Bidwell, Willcutt, & Banich, 2010; Koob & Volkow 2009; Marzi, Regina, & Righi, 2014; Verfaellie & Vasterling, 2009).

## Electronic supplementary material

Below is the link to the electronic supplementary material.

ESM 1 (DOCX 143 kb)

## Footnotes

1 This only includes participants in the “unaided” condition in those studies, which was the condition most comparable to the method we used here. When provided with “aids” (i.e., alternative diversionary thoughts to think about instead of the target), both controls and MDD patients showed far stronger SIF, and differences between the groups were minimized.

2 It should be noted that the two-way interaction between valence and condition in this region was not significant for the MDDs, so we cannot say that individuals in the MDD group modulated this region more for neutral than for negative items.

3 We did not, however, examine functional or effective connectivity between regions. Thus, it is possible that despite similar univariate patterns, connectivity patterns might differ between groups. Future research should directly assess the relations between brain connectivity and suppression.

## References

[CR1] Anderson MC (2004). Neural systems underlying the suppression of unwanted memories. Science.

[CR2] Anderson MC, Green C (2001). Suppressing unwanted memories by executive control. Nature.

[CR3] Anderson MC, Hanslmayr S (2014). Neural mechanisms of motivated forgetting. Trends in Cognitive Sciences.

[CR4] Anderson MC, Huddleston E, Belli RF (2011). Towards a cognitive and neurobiological model of motivated forgetting. True and false recovered memories.

[CR5] Anderson MC, Levy BJ (2009). Suppressing unwanted memories. Current Directions in Psychological Science.

[CR6] Anderson MC, Levy BJ, Benjamin A (2011). On the relationship between interference and inhibition in cognition. Successful remembering and successful forgetting: A festschrift in honor of Robert A. Bjork.

[CR7] Anderson MC, Spellman BA (1995). On the status of inhibitory mechanisms in cognition: Memory retrieval as a model case. Psychological Review.

[CR8] Bär K-J, Wagner G, Koschke M, Boettger S, Boettger MK, Schlösser R, Sauer H (2007). Increased prefrontal activation during pain perception in major depression. Biological Psychiatry.

[CR9] Beck AT (1976). Cognitive therapy and the emotional disorders.

[CR10] Beck AT, Rush AJ, Shaw BF, Emery G (1979). Cognitive therapy of depression.

[CR11] Benoit RG, Anderson MC (2012). Opposing mechanisms support the voluntary forgetting of unwanted memories. Neuron.

[CR12] Benoit RG, Hulbert JC, Huddleston E, Anderson MC (2015). Adaptive top-down suppression of hippocampal activity and the purging of intrusive memories from consciousness. Journal of Cognitive Neuroscience.

[CR13] Bergström ZM, de Fockert JW, Richardson-Klavehn A (2009). ERP and behavioural evidence for direct suppression of unwanted memories. NeuroImage.

[CR14] Bradley MM, Lang PJ (1999). Affective Norms for English Words (ANEW): Stimuli, instruction manual and affective ratings (Technical Report No. C-1).

[CR15] Brewin CR, Gregory JD, Lipton M, Burgess N (2010). Intrusive images in psychological disorders: Characteristics, neural mechanisms, and treatment implications. Psychological Review.

[CR16] Butler AJ, James KH (2010). The neural correlates of attempting to suppress negative versus neutral memories. Cognitive, Affective, & Behavioral Neuroscience.

[CR17] Catarino A, Kupper CS, Werner-Seidler A, Dalgleish T, Anderson MC (2015). Failing to forget: Inhibitory-control deficits compromise memory suppression in posttraumatic stress disorder. Psychological Science.

[CR18] Chrastil ER, Sherrill KR, Hasselmo ME, Stern CE (2015). There and back again: Hippocampus and retrosplenial cortex track homing distance during human path integration. Journal of Neuroscience.

[CR19] Cohen MS (1997). Parametric analysis of fMRI data using linear systems methods. NeuroImage.

[CR20] Cox RW (1996). AFNI: Software for analysis and visualization of functional magnetic resonance neuroimages. Computers and Biomedical Research.

[CR21] Dale AM (1999). Optimal experimental design for event-related fMRI. Human Brain Mapping.

[CR22] Depue BE, Banich MT, Curran T (2006). Suppression of emotional and nonemotional content in memory: Effects of repetition on cognitive control. Psychological Science.

[CR23] Depue BE, Burgess GC, Bidwell LC, Willcutt EG, Banich MT (2010). Behavioral performance predicts grey matter reductions in the right inferior frontal gyrus in young adults with combined type ADHD. Psychiatry Research: Neuroimaging.

[CR24] Depue BE, Curran T, Banich MT (2007). Prefrontal regions orchestrate suppression of emotional memories via a two-phase process. Science.

[CR25] Diener C, Kuehner C, Brusniak W, Ubl B, Wessa M, Flor H (2012). A meta-analysis of neurofunctional imaging studies of emotion and cognition in major depression. NeuroImage.

[CR26] Eickhoff SB, Stephan KE, Mohlberg H, Grefkes C, Fink GR, Amunts K, Zilles K (2005). A new SPM toolbox for combining probabilistic cytoarchitectonic maps and functional imaging data. NeuroImage.

[CR27] Fawcett, J. M., Benoit, R. G., Gagnepain, P., Salman, A., Bartholdy, S., Bradley, C., . . . Anderson, M. C. (2015). The origins of repetitive thought in rumination: Separating cognitive style from deficits in inhibitory control over memory. *Journal of Behavior Therapy and Experimental Psychiatry*, *47*, 1–8. doi:10.1016/j.jbtep.2014.10.009 10.1016/j.jbtep.2014.10.009 PMC4324850 25462596

[CR28] First MB, Dibbon M, Spitzer RL, Williams JB (2004). Structured Clinical Interview for DSM-IV-TR.

[CR29] Friston KJ, Holmes AP, Worsley KJ, Poline JP (1995). Statistical parametric maps in functional imaging: A general linear approach. Human Brain Mapping.

[CR30] Gagnepain P, Henson RN, Anderson MC (2014). Suppressing unwanted memories reduces their unconscious influence via targeted cortical inhibition. Proceedings of the National Academy of Sciences.

[CR31] Glover GH, Law CS (2001). Spiral-in/out BOLD fMRI for increased SNR and reduced susceptibility artifacts. Magnetic Resonance in Medicine.

[CR32] Goeleven E, De Raedt R, Baert S, Koster EHW (2006). Deficient inhibition of emotional information in depression. Journal of Affective Disorders.

[CR33] Gotlib IH, Joormann J (2010). Cognition and depression: Current status and future directions. Annual Review of Clinical Psychology.

[CR34] Gotlib IH, Yue DN, Joormann J (2005). Selective attention in dysphoric individuals: The role of affective interference and inhibition. Cognitive Therapy Research.

[CR35] Grimm, S., Beck, J., Schuepbach, D., Hell, D., Boesiger, P., Bermpohl, F., . . . Northoff, G. (2008). Imbalance between left and right dorsolateral prefrontal cortex in major depression is linked to negative emotional judgment: An fMRI study in severe major depressive disorder. *Biological Psychiatry*, *63*, 369–376. doi:10.1016/j.biopsych.2007.05.033 10.1016/j.biopsych.2007.05.033 17888408

[CR36] Harvey, P.-O., Fossati, P., Pochon, J.-B., Levy, R., Lebastard, G., Lehéricy, S., . . . Dubois, B. (2005). Cognitive control and brain resources in major depression: An fMRI study using the *n*-back task. *NeuroImage*, *26*, 860–869. 10.1016/j.neuroimage.2005.02.048 15955496

[CR37] Hertel PT, Calcaterra G (2005). Intentional forgetting benefits from thought substitution. Psychonomic Bulletin & Review.

[CR38] Hertel PT, Gerstle M (2003). Depressive deficits in forgetting. Psychological Science.

[CR39] Hertel PT, Mahan A (2008). Depression-related differences in learning and forgetting responses to unrelated cues. Acta Psychologica.

[CR40] Hulbert JC, Henson RN, Anderson MC (2016). Inducing amnesia through systemic suppression. Nature Communications.

[CR41] Ingram RE (1984). Toward an information-processing analysis of depression. Cognitive Therapy Research.

[CR42] Joormann J (2004). Attentional bias in dysphoria: The role of inhibitory processes. Cognition and Emotion.

[CR43] Joormann J, Gotlib IH (2008). Updating the contents of working memory in depression: Interference from irrelevant negative material. Journal of Abnormal Psychology.

[CR44] Joormann J, Hertel PT, Brozovich F, Gotlib IH (2005). Remembering the good, forgetting the bad: Intentional forgetting of emotional material in depression. Journal of Abnormal Psychology.

[CR45] Joormann J, Hertel PT, LeMoult J, Gotlib IH (2009). Training forgetting of negative material in depression. Journal of Abnormal Psychology.

[CR46] Joormann J, Nee DE, Berman MG, Jonides J, Gotlib IH (2010). Interference resolution in major depression. Cognitive, Affective, & Behavioral Neuroscience.

[CR47] Joormann J, Tran TB (2009). Rumination and intentional forgetting of emotional material. Cognition and Emotion.

[CR48] Karpicke JD (2012). Retrieval-based learning: Active retrieval promotes meaningful learning. Current Directions in Psychological Science.

[CR49] Kircanski K, Joormann J, Gotlib IH (2012). Cognitive aspects of depression. Wiley Interdisciplinary Reviews: Cognitive Science.

[CR50] Koob GF, Volkow ND (2009). Neurocircuitry of addiction. Neuropsychopharmacology.

[CR51] Levens SM, Gotlib IH (2010). Updating positive and negative stimuli in working memory in depression. Journal of Experimental Psychology: General.

[CR52] Levy BJ, Anderson MC (2008). Individual differences in the suppression of unwanted memories: The executive deficit hypothesis. Acta Psychologica.

[CR53] Levy BJ, Anderson MC (2012). Purging of memories from conscious awareness tracked in the human brain. Journal of Neuroscience.

[CR54] Marzi T, Regina A, Righi S (2014). Emotions shape memory suppression in trait anxiety. Frontiers in Psychology.

[CR55] Mathews A, MacLeod C (2005). Cognitive vulnerability to emotional disorders. Annual Review of Clinical Psychology.

[CR56] Matt GE, Vázquez C, Campbell WK (1992). Mood-congruent recall of affectively toned stimuli: A meta-analytic review. Clinical Psychology Review.

[CR57] Nolen-Hoeksema S (2000). The role of rumination in depressive disorders and mixed anxiety/depressive symptoms. Journal of Abnormal Psychology.

[CR58] Paz-Alonso PM, Bunge SA, Anderson MC, Ghetti S (2013). Strength of coupling within a mnemonic control network differentiates those who can and cannot suppress memory retrieval. Journal of Neuroscience.

[CR59] Talairach J, Tournoux P (1998). Co-planar stereotaxic atlas of the human brain: 3-dimensional proportional system. An approach to cerebral imaging.

[CR60] Teasdale JD (1983). Negative thinking in depression: Cause, effect, or reciprocal relationship?. Advances in Behaviour Research and Therapy.

[CR61] Tulving E, Thomson DM (1973). Encoding specificity and retrieval processes in episodic memory. Psychological Review.

[CR62] Verfaellie M, Vasterling JJ, Shiromani PJ, Keane TM, LeDoux JE (2009). Memory in PTSD: A neurocognitive approach. Post-traumatic stress disorder: Basic science and clinical practice.

[CR63] Wagner, G., Sinsel, E., Sobanski, T., Köhler, S., Marinou, V., Mentzel, H.-J., . . . Schlösser, R. G. (2006). Cortical inefficiency in patients with unipolar depression: An event-related fMRI study with the Stroop task. *Biological Psychiatry*, *59*, 958–965. doi:10.1016/j.biopsych.2005.10.025 10.1016/j.biopsych.2005.10.025 16458263

[CR64] Walter H, Wolf RC, Spitzer M, Vasic N (2007). Increased left prefrontal activation in patients with unipolar depression: An event-related, parametric, performance-controlled fMRI study. Journal of Affective Disorders.

[CR65] Williams JMG, Watts F, MacLeod C, Mathews A (1997). Cognitive psychology and emotional disorders.

[CR66] Woo C-W, Krishnan A, Wager TD (2014). Cluster-extent based thresholding in fMRI analyses: Pitfalls and recommendations. NeuroImage.

[CR67] Xiong J, Gao JH, Lancaster JL, Fox PT (1995). Clustered pixels analysis for functional MRI activation studies of the human brain. Human Brain Mapping.

