# Supplementary material for: Cognitive and neural consequences of memory suppression in major depressive disorder
Source: Cogn Affect Behav Neurosci. 2016 Sep 20;17(1):77–93. doi: 10.3758/s13415-016-0464-x (PMC5272890; doi:10.3758/s13415-016-0464-x)
Supplement: Supplementary file 1 — (DOCX 143 kb) [file 13415_2016_464_MOESM1_ESM.docx]

**Supplemental Method**

We also administered a number of questionnaires to each participant. The Ruminative Response Styles (RRS; Treynor et al. 2003) questionnaire assesses self-reported tendency to ruminate (trait), i.e., repetitive negative thinking focused on the self. The RRS is the most commonly used questionnaire to assess depressive rumination (Whitmer and Gotlib 2012), and includes two 5-item subscales: reflection and brooding. The reflection subscale measures emotionally neutral pondering, while the brooding subscale measures self-critical “moody” pondering. Subscale scores were computed as the sum of the respective subscale items. The Beck Depression Inventory (BDI; Beck et al. 1996) is a self-report measure of depression severity and has been widely used in to assess levels of depression symptoms. The BDI assesses core features of MDD including levels of sadness, hopelessness, anhedonia, guilt, and disappointment. We used the 28-item Positive and Negative Affect Schedule (PANAS; Watson et al. 1988) to measure levels of positive and negative affect. This questionnaire asks participants to rate the extent to which they are experiencing a given feeling or emotion (on a five-point Likert scale from very slightly to extremely). Positive and negative affect were measured separately as the sum of the 14 respective items.

**Supplemental Results**

As expected, MDD participants scored lower than CTL participants on positive affect (*t*(30) = 4.55, *p* < 0.001), and higher on negative affect (*t*(30) = 5.19, *p* < 0.001), and the RRS reflection (*t*(30) = 6.17, *p* < 0.001) and brooding (*t*(30) = 8.59, *p* < 0.001;) subscales. See Table S1 for descriptive statistics for each group for each measure.

Prior studies have investigated whether activity in specific ROIs, particularly within the prefrontal or medial temporal lobes and the amygdala, correlates with behavioral measures of forgetting (e.g., Anderson 2004; Depue et al. 2007; Levy and Anderson 2012). We took the same approach here by using behavioral suppression-induced forgetting scores (Baseline recall - No Think recall, thus more positive scores indicate greater forgetting), and examining whether these scores correlated with activity in any of the three ROIs identified earlier (right middle frontal gyrus [MFG], left hippocampus/amygdala, or right hippocampus/amygdala). Following earlier studies, we subtracted activity during Think trials from activity during No Think trials, so higher scores indicate greater activity during the attempt to suppress a memory. Importantly, we restricted these analyses within a given valence (i.e., behavior on neutral trials was compared only to brain activity on neutral trials). There were no significant correlations between these behavioral and brain measures (Table S2). We also performed a similar analysis for our behavioral measure of how much the Think facilitated memory for practiced items (Think recall – Baseline recall) and again observed no significant correlations (Table S2).

Prior studies have also reported correlations in activity between brain regions (e.g., Anderson 2004; Depue et al. 2007) therefore, we also compared activity between these regions. Specifically, we looked for evidence that increased activity in the right MFG during suppression would be related to decreased activity in either of the regions we expect might be targeted by inhibition (i.e., the regions in the hippocampus/amygdala). Again, none of these correlations were significant (Table S3).

We also conducted a set of exploratory analyses based on other clinical and psychological measures collected for each participant. In these analyses we examined whether differences in depression severity, affect, or rumination were related to brain activity. We looked for any significant correlations between activity in our three ROIs and each of the following behavioral measures: BDI, positive affect, negative affect, and rumination. Importantly, because there were large group differences on each of these measures (i.e., on these variables the two groups were largely non-overlapping), we only assessed these correlations within each group. Although we did find several significant correlations, it is important to note that none of them would survive a correction for multiple comparisons. First, we found a correlation between higher BDI scores and less activity in the left hippocampus/amygdala during negative No Think trials relative to Think trials (*r* = -0.62, *p* < 0.05). Interestingly, a similar (but nonsignificant) pattern was observed in the right hemisphere (*r* = -0.43, *p* = 0.10). We also found that in MDD participants, higher RRS brooding subscale scores were correlated with less recruitment of the right hippocampus/amygdala during negative No Think trials relative to negative Think trials (*r* = -0.60, *p* < 0.05), while a similar statistical trend was observed in left hippocampus/amygdala (*r*  = -0.47, *p* = 0.07). Finally, there was also a significant positive correlation between levels of negative affect and left hippocampus/amygdala activity during Neutral trials (*r* = 0.51, *p* < 0.05). Comprehensive results are included in Table S4. Given the exploratory nature of these analyses, we are reluctant to draw strong conclusions. With that said these observations are potentially interesting and should be studied further in future research.

**Supplemental References**

Anderson, M. C. (2004). Neural Systems Underlying the Suppression of Unwanted Memories. *Science*, *303*(5655), 232–235.

Beck, A. T., Steer, R. A., & Brown, G. K. (1996). *Manual for the Beck Depression Inventory-II*. San Antonio, TX: Psychological Corporation.

Depue, B. E., Curran, T., & Banich, M. T. (2007). Prefrontal Regions Orchestrate Suppression of Emotional Memories via a Two-Phase Process. *Science*, *317*(5835), 215–219.

Levy, B. J., & Anderson, M. C. (2012). Purging of Memories from Conscious Awareness Tracked in the Human Brain. *Journal of Neuroscience*, *32*(47), 16785–16794. doi:10.1523/JNEUROSCI.2640-12.2012

Treynor, W., Gonzalez, R., & Nolen-Hoeksema, S. (2003). Rumination Reconsidered: A Psychometric Analysis. *Cognitive Therapy Research*, *27*(3), 247–259.

Watson, D., Clark, L. A., & Tellegen, A. (1988). Development and validation of brief measures of positive and negative affect: the PANAS scales. *Journal of personality and social psychology*, *54*(6), 1063–1070.

Whitmer, A. J., & Gotlib, I. H. (2012). An Attentional Scope Model of Rumination. *Psychological Bulletin*, *139*(5), 1036–1061.

**Table S1.** Psychological and Clinical Characteristics by Group.

|  | **MDD** | | **CTL** | |
| --- | --- | --- | --- | --- |
|  | ***M*** | ***M*** | ***M*** | ***SD*** |
| **Positive Affect*** | 23.1 | 38.0 | 38.0 | 7.3 |
| **Negative Affect*** | 30.7 | 16.6 | 16.6 | 10.7 |
| **RRS Reflection** | 14.2 | 7.1 | 7.1 | 3.3 |
| **RRS Brooding** | 15.4 | 6.6 | 6.6 | 3.4 |

CTL = control participants; MDD = Major Depressive Disorder particpants; *M* = mean; *SD* = standard deviation; * = as measured by the Positive and Negative Affect Schedule (PANAS); RRS = Ruminative Response Styles questionnaire subscale**.**

**Table S2.** Correlations between Behavioral Memory Measures and Brain Activity in the Regions of Interests. Pearson correlation coefficients are reported across all individuals. Coefficients greater than or equal to 0.35 or less than or equal to -0.35 are associated with *p*-values less than 0.05 (uncorrected for multiple comparisons).

|  | | **Right Middle Frontal Gyrus (MFG)** | | **Right Hippocampus/Amygdala** | | **Left Hippocampus/Amygdala** | |
| --- | --- | --- | --- | --- | --- | --- | --- |
|  |  | **Neutral** | **Negative** | **Neutral** | **Negative** | **Neutral** | **Negative** |
| **Suppression-induced forgetting (SIF)** | **SP&IP** | -0.08 | -0.27 | -0.06 | 0.11 | -0.04 | 0.15 |
|  | **SP** | 0.00 | -0.24 | -0.09 | -0.03 | -0.08 | -0.06 |
|  | **IP** | -0.10 | -0.18 | -0.01 | 0.15 | 0.00 | 0.22 |
| **Retrieval-induced facilitation** | **SP&IP** | 0.23 | -0.15 | 0.00 | -0.11 | -0.16 | 0.07 |
|  | **SP** | 0.15 | -0.12 | 0.09 | -0.07 | 0.01 | 0.00 |
|  | **IP** | 0.19 | -0.11 | -0.04 | -0.08 | -0.19 | 0.07 |

SP = same probe; IP = independent probe.

**Table S3.** Correlations between Activity in the Right MFG Region of Interest and Activity in the Hippocampus/Amygdala Regions of Interest. Pearson correlation coefficients are reported across groups and within each group. Across all participants, coefficients greater than or equal to 0.35 or less than or equal to -0.35 are associated with *p*-values less than 0.05 (uncorrected for multiple comparisons). Within groups, coefficients greater than or equal to 0.50 or less than or equal to -0.50 are associated with *p*-values less than 0.05 (uncorrected for multiple comparisons).

|  | **Right Hippocampus/Amygdala** | | **Left Hippocampus/Amygdala** | |
| --- | --- | --- | --- | --- |
|  | **Neutral** | **Negative** | **Neutral** | **Negative** |
| **All participants** | 0.09 | 0.25 | 0.09 | 0.26 |
| **MDD participants** | 0.29 | 0.17 | 0.46 | 0.30 |
| **CTL participants** | 0.14 | 0.24 | 0.11 | 0.24 |

MDD = Major Depressive Disorder; CTL = control.

**Table S4.** Exploratory Correlations between Brain Activity in the Regions of Interest and Behavioral Measures. Pearson correlation coefficients are reported within each group. Coefficients greater than or equal to 0.50 or less than or equal to -0.50 are associated with *p*-values less than 0.05 (uncorrected for multiple comparisons).

|  | **Right Middle Frontal Gyrus (MFG)** | | **Right Hippocampus/Amygdala** | | **Left Hippocampus/Amygdala** | |
| --- | --- | --- | --- | --- | --- | --- |
| **MDD participants** | **Neutral** | **Negative** | **Neutral** | **Negative** | **Neutral** | **Negative** |
| *BDI* | 0.35 | -0.31 | 0.32 | -0.43 | 0.45 | -0.62 |
| *PANAS negative* | 0.47 | -0.17 | 0.36 | -0.43 | 0.51 | -0.48 |
| *PANAS positive* | 0.22 | 0.07 | -0.08 | -0.07 | 0.11 | 0.06 |
| *RRS (Brooding)* | 0.32 | 0.05 | 0.12 | -0.60 | 0.35 | -0.47 |
| *RRS (Reflection)* | 0.24 | -0.09 | 0.04 | -0.29 | 0.17 | -0.39 |
| **CTL participants** | **Neutral** | **Negative** | **Neutral** | **Negative** | **Neutral** | **Negative** |
| *BDI* | -0.24 | 0.04 | 0.36 | 0.22 | -0.21 | 0.42 |
| *PANAS negative* | 0.23 | -0.03 | 0.26 | 0.23 | 0.23 | 0.44 |
| *PANAS positive* | -0.22 | 0.38 | -0.12 | 0.04 | 0.36 | 0.17 |
| *RRS (Brooding)* | 0.04 | -0.01 | 0.39 | 0.26 | 0.24 | 0.00 |
| *RRS (Reflection)* | 0.34 | 0.31 | 0.37 | 0.38 | 0.12 | 0.11 |

MDD = Major Depressive Disorder; CTL = control; BDI = Beck Depression Inventory; PANAS = Positive and Negative Affect Schedule; RRS = Ruminative Response Styles questionnaire.
